# Metabolic, Cardiac, and Hemorheological Responses to Submaximal Exercise under Light and Moderate Hypobaric Hypoxia in Healthy Men

**DOI:** 10.3390/biology11010144

**Published:** 2022-01-15

**Authors:** Hun-Young Park, Jeong-Weon Kim, Sang-Seok Nam

**Affiliations:** 1 Physical Activity and Performance Institute, Konkuk University, 120 Neungdong-ro, Gwangjin-gu, Seoul 05029, Korea; parkhy1980@konkuk.ac.kr; 2 Department of Sports Medicine and Science, Graduate School, Konkuk University, 120 Neungdong-ro, Gwangjin-gu, Seoul 05029, Korea; 3 Graduate School of Professional Therapy, Gachon University, 1342 Seongnam-daero, Sujeong-gu, Seongnam-si 13120, Korea; zeezone@gachon.ac.kr; 4 Taekwondo Research Institute of Kukkiwon, 32 Teheran7gil, Gangnam-gu, Seoul 06130, Korea

**Keywords:** metabolic parameters, cardiac function, hemorheological parameters, hypoxia

## Abstract

**Simple Summary:**

The lower atmospheric partial pressure of oxygen under hypobaric hypoxia decreases oxygen saturation and arteriovenous oxygen difference. Exercise under hypoxia decreases arterial oxygen saturation, which reduces the ability to deliver oxygen to active muscles and consequently worsens aerobic capacity and exercise performance. Previous studies on metabolic and cardiac responses to submaximal exercise under hypoxia have been well documented, but information on hemorheological responses is relatively insufficient. In this regard, a review of hemorheological responses to exercise under hypoxia could provide further information on reduced aerobic capacity and exercise performance caused by acute hypoxia. We conducted a randomized crossover trial to compare the effects of acute exercise under light and moderate hypobaric hypoxia versus normoxia on metabolic parameters, cardiac function, and hemorheological properties in healthy men. The main findings of our study revealed that endurance submaximal exercise under light (596 mmHg, simulated 2000 m) and moderate (526 mmHg, simulated 3000 m) hypoxia induced greater metabolic and cardiac responses than exercise under normoxia. However, exercise under hypobaric hypoxia did not affect hemorheological properties, including erythrocyte deformability and aggregation. These results can be used as basic data for understanding hemorheological responses in light and moderate hypobaric hypoxia.

**Abstract:**

We compared the effects of metabolic, cardiac, and hemorheological responses to submaximal exercise under light hypoxia (LH) and moderate hypoxia (MH) versus normoxia (N). Ten healthy men (aged 21.3 ± 1.0 years) completed 30 min submaximal exercise corresponding to 60% maximal oxygen uptake at normoxia on a cycle ergometer under normoxia (760 mmHg), light hypoxia (596 mmHg, simulated 2000 m altitude), and moderate hypoxia (526 mmHg, simulated 3000 m altitude) after a 30 min exposure in the respective environments on different days, in a random order. Metabolic parameters (oxygen saturation (S_P_O_2_), minute ventilation, oxygen uptake, carbon dioxide excretion, respiratory exchange ratio, and blood lactate), cardiac function (heart rate (HR), stroke volume, cardiac output, and ejection fraction), and hemorheological properties (erythrocyte deformability and aggregation) were measured at rest and 5, 10, 15, and 30 min after exercise. S_P_O_2_ significantly reduced as hypoxia became more severe (MH > LH > N), and blood lactate was significantly higher in the MH than in the LH and N groups. HR significantly increased in the MH and LH groups compared to the N group. There was no significant difference in hemorheological properties, including erythrocyte deformability and aggregation. Thus, submaximal exercise under light/moderate hypoxia induced greater metabolic and cardiac responses but did not affect hemorheological properties.

## 1. Introduction

The Olympic Games in 1968 were held at high altitudes in Mexico City. Since then, the effects of hypoxia on exercise performance have received considerable attention [1]. Exercise training under hypoxia has been widely accepted as a useful modality for improving athletic performance, and experimental evidence has been accumulated regarding the efficacy of hypoxic training [2,3,4,5,6]. Exercise under hypoxia decreases arterial oxygen saturation, which reduces the ability to deliver oxygen to active muscles [7]. This phenomenon augments the heart rate (HR) to supply blood flow, carbohydrate substrate mobilization with high oxygen utilization efficiency, and blood lactate levels [8,9,10]. Exercise under hypoxia is also known to achieve a greater metabolic effect and cardiac stress while maintaining the same mechanical stimulus compared with exercise under normoxia [10,11]. Therefore, sustained exercise under hypoxia might be beneficial for enhancing performance, promoting metabolic adaptation in the muscle, and improving cardiac function [2,3,12,13,14]. However, the mechanisms underlying these adaptive phenomena are not clearly understood.

Exercise under hypoxia has shown an additive improving effect on vascular function and microcirculation, arterial stiffness, and blood flow within the skeletal muscle vascular beds [10,11,15]. Hypoxia improves the endothelial-derived nitric-oxide-mediated mechanism primarily involved in the vasodilation of muscular arteries, and consequently enhances the ability of the vasculature to supply the blood to the skeletal muscle, in addition to enhancing the aerobic capacity [16,17]. In addition, chronic exercise training under hypoxia positively induces the secretion of various vasodilators (e.g., adenosine, prostaglandin, and nitric oxide) and vascular endothelial growth factor, which leads to the improvement of vasodilation and microcirculation, as well as the reduction of arterial stiffness [18,19,20].

Hemorheological properties such as erythrocyte deformability and aggregation affect the oxygen delivery capacity of active skeletal muscles [21,22]. The blood flow through capillaries, which are smaller in diameter than erythrocytes and microcirculation, is accompanied by erythrocyte deformability [21]. Erythrocyte deformability is an energy-dependent process, and healthy erythrocytes with high deformability show a high correlation with oxygen-carrying capacity and aerobic capacity [21,22,23]. Erythrocyte aggregation also refers to the binding of two erythrocytes, such as one-dimensional rouleaux formation. Elevated erythrocyte aggregation is frequently observed in sedentary/overweight/obese people and those with vascular diseases and diabetes mellitus. The hyper-aggregation of erythrocytes may cause flow resistance in microcirculation and yield poor oxygen delivery and aerobic capacity [21,23,24,25]. A previous study reported that a 12-week Pilates intervention under moderate hypoxia (inspired oxygen fraction (F_i_O_2_) = 14.5%, simulated 3000 m) improved erythrocyte deformability and aggregation in women with obesity compared with a Pilates intervention under normoxia [12]. These results show that exercise under hypoxia may enhance aerobic capacity and microcirculation by improving hemorheological properties.

However, studies on hypoxia-induced hemorheological responses during exercise are limited. A review of hemorheological responses to exercise under hypoxia could provide further information on reduced aerobic capacity and exercise performance caused by acute hypoxia. Therefore, it is a valuable and important task to elucidate the relationship between hypoxia and aerobic performance by investigating the effect of hypoxia on hemorheological properties such as erythrocyte deformability and aggregation, which is one of the important determinants of oxygen-delivering capacity.

We conducted a randomized crossover trial to compare the effects of acute exercise under light and moderate hypobaric hypoxia versus normoxia on metabolic parameters, cardiac function, and hemorheological properties in healthy men. We hypothesized that endurance submaximal exercise under light and moderate hypobaric hypoxia would induce greater exercise-mediated alterations in metabolic parameters, cardiac function, and hemorheological properties than exercise under normoxia.

## 2. Materials and Methods

### 2.1. Participants

**Figure 1:**
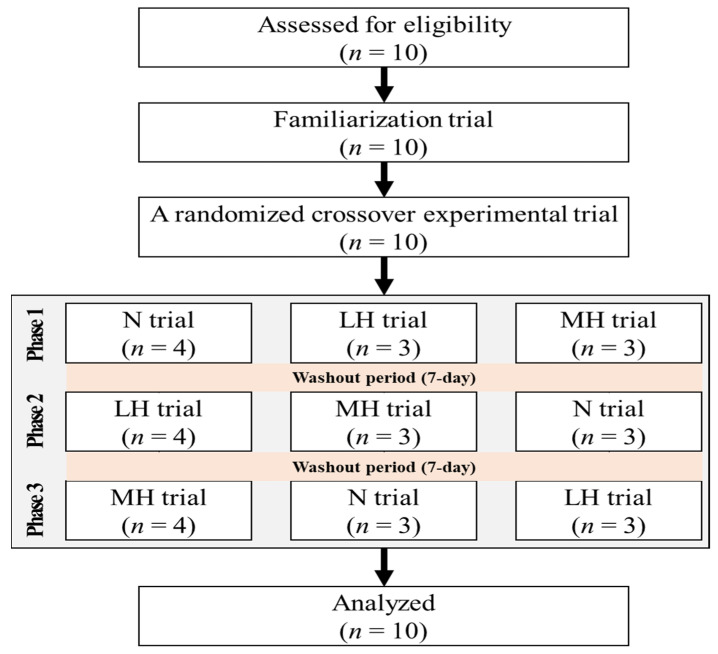
The flow diagram of the consolidated standards of reporting trial. N: endurance submaximal exercise under normoxia; LH: endurance submaximal exercise under light hypoxia (596 mmHg, simulated 2000 m); MH: endurance submaximal exercise under moderate hypoxia (526 mmHg, simulated 3000 m).

Ten healthy men, aged 20 to 25 years old, who were nonsmokers and had no history of musculoskeletal, cardiovascular, or pulmonary diseases (age, 21.3 ± 1.0 years; height, 176.9 ± 1.4 cm; fat-free mass, 35.5 ± 1.4 kg; fat mass, 11.3 ± 1.6 kg; percent body fat, 15.5 ± 1.6%) were recruited. They had not participated in any exercise program under hypoxic conditions in the previous 6 months. The Consolidated Standards of Reporting Trials (CONSORT) flow diagram is shown in Figure 1. The participants received information about the purpose and process of this study and provided informed consent prior to the start of the study. This study was approved by the Institutional Review Board of Kyung Hee University (KHSIRB 2015-020) in Korea and was conducted in accordance with the Declaration of Helsinki.

### 2.2. Study Design

**Figure 2:**
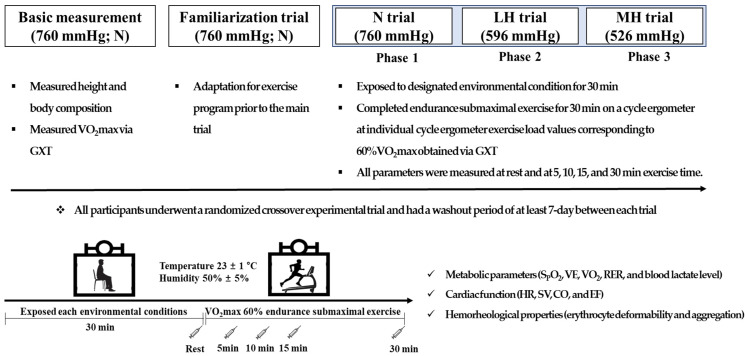
Study design. N: endurance submaximal exercise under normoxia; LH: endurance submaximal exercise under light hypoxia (596 mmHg, simulated 2000 m); MH: endurance submaximal exercise under moderate hypoxia (526 mmHg, simulated 3000 m); VO_2_max: maximal oxygen uptake; GXT: graded exercise test; S_p_O_2_: oxygen saturation; VE: minute ventilation; VO_2_: oxygen uptake; VCO_2_: carbon dioxide excretion; RER: respiratory exchange ratio; HR: heart rate; SV: stroke volume; CO: cardiac output; EF: ejection fraction.

The design of the present study is illustrated in Figure 2. All participants visited the laboratory five times during the experimental period. During the first visit, all participants fasted for more than 8 h, and after stabilization, height and body composition were measured in the morning. Then, they performed a graded exercise test (GXT) to assess 60% maximal oxygen uptake (VO_2_max) and determine the exercise intensity during submaximal exercise under normoxia, light hypobaric hypoxia, and moderate hypobaric hypoxia. On the second visit, they underwent a familiarization trial at sea level (760 mmHg) prior to the main trial for adaptation to the exercise program. On the third, fourth, and fifth occasions, the participants underwent randomized crossover experimental trials under normoxia (760 mmHg; N), light hypoxia (596 mmHg, simulated 2000 m; LH), and moderate hypoxia (526 mmHg, simulated 3000 m; MH). All participants had a washout period of at least 7 days between all trials. The order of the conditions for each experimental trial was randomized, and each participant underwent the experimental protocol at the same time at each visit. However, the participants did not undergo blinded experiments under environmental conditions.

During all experimental trials, all participants were exposed to the designated environmental condition for 30 min; then, they completed endurance submaximal exercise for 30 min on a cycle ergometer (Aerobike 75XLII, Konami Corporation, Tokyo, Japan) at individual cycle ergometer exercise load values corresponding to 60% VO_2_max obtained via GXT. During the endurance submaximal exercise session, metabolic parameters (oxygen saturation (S_p_O_2_), minute ventilation (VE), oxygen uptake (VO_2_), carbon dioxide excretion (VCO_2_), respiratory exchange ratio (RER), and blood lactate level), cardiac function (HR, stroke volume (SV), cardiac output (CO), and ejection fraction (EF)), and hemorheological properties (erythrocyte deformability and aggregation) were measured at rest and at 5, 10, 15, and 30 min.

All experiments were performed in a 6.5 × 7.5 × 3 m (width × length × height) environmental chamber (Submersible Systems, Huntington Beach, CA, USA) at a temperature of 23 ± 1 °C and humidity of 50 ± 5% regulated by an environmental control chamber.

### 2.3. Measurement

Body composition (i.e., height, weight, fat-free mass, and percentage body fat) was measured after fasting for more than 8 h using a bioelectrical impedance analysis device (Inbody 770; Inbody, Seoul, Korea).

Metabolic parameters (e.g., S_P_O_2_, VE, VO_2_, VCO_2_, RER, and blood lactate level) were measured at rest and at 5, 10, 15, and 30 min of exercise time. The S_P_O_2_ was measured using a Radical-7 pulse oximeter (Masimo, Irvine, CA, USA). VE, VO_2_, VCO_2_, and RER were measured using the K4B2 auto metabolism analyzer (Cosmed, Rome, Italy) and a breathing valve in the form of a facemask during the 30 min endurance submaximal exercise. Blood lactate levels were analyzed using a YSI-1500 lactate analyzer (YSI Inc., Yellow Springs, OH, USA).

Cardiac functions, including HR, SV, CO, and EF, were noninvasively assessed at rest and at 5, 10, 15, and 30 min of exercise time using a thoracic bioelectrical impedance device (PhysioFlow PF-05, Paris, France) during the 30 min endurance submaximal exercise.

Hemorheological properties, erythrocyte deformability, and aggregation were analyzed as suggested in previous studies [21,24]. A 20G catheter (Sewoonmedical Co., Ltd., Cheonan-si, Chungcheongnam-do, Korea) was inserted in the forearm vein and connected using a three-way extension line (Sewoonmedical Co., Ltd., Cheonan-si, Chungcheongnam-do, Korea). The blood (6 mL) was collected in two K3-ethylenediaminetetraacetic acid (EDTA) tubes (Greiner Bio-One Ltd., Chon Buri, Thailand) at rest and during exercise (5, 10, 15, and 30 min). We refrained from using a tourniquet as much as possible because the use of a tourniquet during venous blood sampling can decrease erythrocyte deformability and increase erythrocyte aggregation [25]. However, if necessary, the use of a tourniquet was limited to 5 s. All blood samples were analyzed within 3 h of collection at room temperature (25 °C) using a Rheoscan-D (Rheo Meditech Inc., Seongbuk-gu, Seoul, Korea) [12,21]. Erythrocyte deformability was evaluated using the elongation index (EI) by the following process: after transferring the sample to a 2 mL microfuge tube, it was diluted in 700 μL of 5.5% polyvinylpyrrolidone (360 kDa) dissolved in 1 mmol phosphate-buffered saline (pH 7.4; osmolality: 300 mOsmol/kg) in a K3-EDTA tube (Greiner Bio-one, Chon Nuri, Thailand). Thereafter, this solution (0.5 mL) was analyzed using a D-test kit according to the manufacturer’s instructions (Rheo Meditech Inc., Seongbuk-gu, Seoul, Korea). The accuracy of erythrocyte EI was measured using a Lineweaver–Burk plot model [21,24]. Erythrocyte aggregation was evaluated using the aggregation index (AI) by the following process: 8 μL of the whole-blood sample was analyzed using an A-test kit according to the manufacturer’s instructions (Rheo Meditech Inc., Seongbuk-gu, Seoul, Korea) [12,21].

### 2.4. Statistical Analysis

All statistical analyses were conducted using SPSS version 25.0 (IBM Corp., Armonk, NY, USA) for Windows. Data are presented as mean ± standard deviation. To determine the sample size, we focused on identifying meaningful differences in S_P_O_2_ during submaximal exercise, as previously suggested [26]. An a priori power analysis, which was performed using G-power, indicated that a minimum sample size of 8 participants would be required to provide 80% power at an α-level of 0.05. Anticipating a dropout rate > 10%, we aimed for a starting sample size of 10 participants. The normality of the distribution of all outcome variables was verified using the Shapiro–Wilk W-test prior to parametric tests. A two-way analysis (trial × time) of variance (ANOVA) with repeated measures was used to assess the presence of interactions (trial × time) and main effects (trial or time). When ANOVA revealed a significant interaction or main effect within the trial, a Bonferroni post-hoc test was used to identify within-trial differences at each time point. The level of significance was set a priori at *p* < 0.05.

## 3. Results

### 3.1. Metabolic Parameters

**Figure 3:**
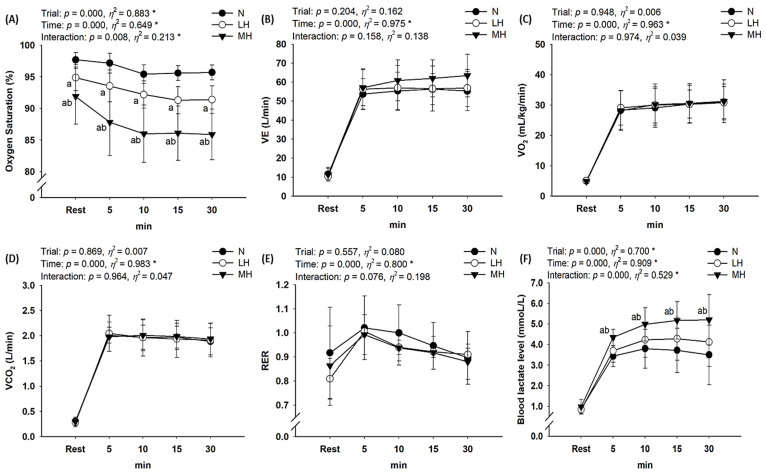
Changes in metabolic parameters at rest and during endurance submaximal exercise. (**A**) S_P_O_2_, (**B**) VE, (**C**) VO_2_, (**D**) VCO_2_, (**E**) RER, (**F**) blood lactate level. S_P_O_2_: oxygen saturation; VE: minute ventilation; VO_2_: oxygen uptake; VCO_2_: carbon dioxide excretion; RER: respiratory exchange ratio; N: endurance submaximal exercise under normoxia; LH: endurance submaximal exercise under light hypoxia (596 mmHg, simulated 2000 m); MH: endurance submaximal exercise under moderate hypoxia (526 mmHg, simulated 3000 m). The bars indicate the mean ± S.D. *: Significant interaction or main effect; a: significant difference vs. normoxia; b: significant difference vs. light hypoxia.

As shown in Figure 3, there was no significant interaction between VE, VO_2_, VCO_2_, and RER; however, S_P_O_2_ (*p* = 0.008, *η*^2^ = 0.213) and blood lactate level (*p* < 0.001, *η*^2^ = 0.529) showed a significant interaction. Post-hoc analysis indicated the significant reduction in S_P_O_2_ as the hypoxia became more severe in the order of MH > LH > N (main effect for trial, *p* < 0.001, *η*^2^ = 0.883); blood lactate levels were significantly higher in the MH group than in LH and N groups (main effect for trial, *p* < 0.001, *η*^2^ = 0.700).

### 3.2. Cardiac Function

**Figure 4:**
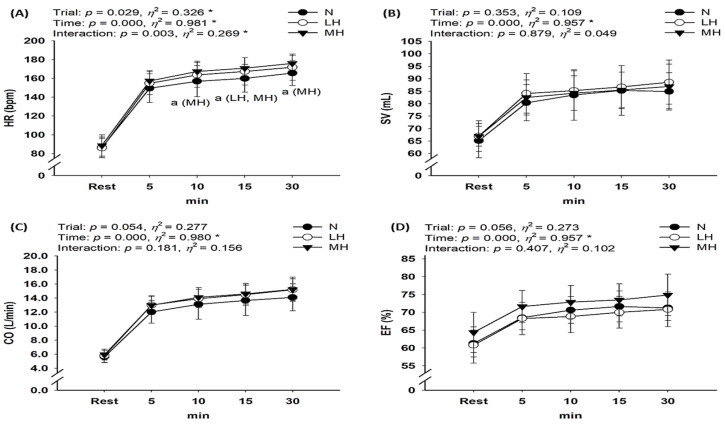
Changes in the cardiac function at rest and during endurance submaximal exercise. (**A**) HR, (**B**) SV, (**C**) CO, (**D**) EF. HR: heart rate; SV: stroke volume; CO: cardiac output; EF: ejection fraction; N: endurance submaximal exercise under normoxia; LH: endurance submaximal exercise under light hypoxia (596 mmHg, simulated 2000 m); MH: endurance submaximal exercise under moderate hypoxia (526 mmHg, simulated 3000 m). The bars indicate the mean ± S.D. *: Significant interaction or main effect; a: significant difference vs. normoxia.

Figure 4 presents the cardiac function results during submaximal exercise between different groups. No significant interaction was observed between SV, CO, and EF; however, HR showed a significant interaction (*p* < 0.001, *η*^2^ = 0.529). Post-hoc analysis indicated that HR was significantly higher for MH and LH groups than for the N group (main effect for trial, *p* = 0.029, *η*^2^ = 0.326). In addition, CO showed an increased tendency in the MH group compared with N and LH groups (*p* = 0.054, *η*^2^ = 0.277).

### 3.3. Hemorheological Properties

**Figure 5:**
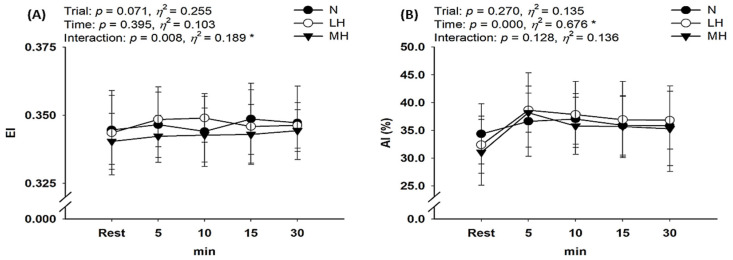
Changes in hemorheological properties at rest and during endurance submaximal exercise. (**A**) Erythrocyte deformability (EI) and (**B**) Erythrocyte aggregation (AI). EI: elongation index; AI: aggregation index; N: endurance submaximal exercise under normoxia; LH: endurance submaximal exercise under light hypoxia (596 mmHg, simulated 2000 m); MH: endurance submaximal exercise under moderate hypoxia (526 mmHg, simulated 3000 m). The bars indicate the mean ± S.D. *: Significant interaction or main effect.

As shown in Figure 5, there was no significant interaction with erythrocyte aggregation; however, erythrocyte deformability showed a significant interaction (*p* = 0.008, *η*^2^ = 0.189). Post-hoc analysis indicated no significant difference in erythrocyte deformability between the groups (main effect for trial, *p* = 0.071, *η*^2^ = 0.255).

## 4. Discussion

According to our hypothesis, endurance submaximal exercise under light and moderate hypobaric hypoxia induces greater responses in some metabolic parameters (e.g., S_P_O_2_ and blood lactate level) and cardiac function (e.g., HR) than exercise under normoxia. However, there were no significant changes in VE, VO_2_, VCO_2_, RER, SV, CO, or EF between LH or MH groups and the N group. In addition, exercise under hypoxia did not affect hemorheological properties, including erythrocyte deformability and aggregation, compared with exercise under normoxia.

The lower atmospheric partial pressure of oxygen under hypobaric hypoxia decreases S_p_O_2_ and the arteriovenous oxygen difference and worsens the aerobic capacity and exercise performance [1,7,21,26]. Hypoxia also reduces oxygen availability in active muscles owing to reduction in the oxygen delivery capacity of the blood, resulting in decreased VO_2_ during endurance submaximal exercise at the same relative intensity (same mechanical load), as well as a decrease in VO_2_max [26,27]. In the present study, we confirmed the significant decrease in S_p_O_2_ as hypoxia became more severe (MH > LH > N). However, we did not observe any significant differences in VO_2_ during endurance submaximal exercise between all environmental conditions. It is likely that the energy consumption was the same because the mechanical load was similar during endurance submaximal exercise under all environmental conditions [28,29,30]. Several previous studies have reported no significant differences in VO_2_ during submaximal exercise at the same mechanical load between normoxia and hypoxia. There was no difference in VO_2_ during submaximal exercise between normoxia and hypoxia conditions, which may be explained by changes in cardiac function, especially the increase in HR and CO in response to reduced oxygen availability in active muscles following reduction in the oxygen delivery capacity of the blood [7,10,26]. Moon et al. [26] evaluated the effects of hypoxia (F_i_O_2_ = 16.5%, 14.5%, 12.8%, and 11.2%) versus normoxia (F_i_O_2_ = 20.9%) on metabolic parameters and cardiac function during constant load (116.7 ± 20.1 watts and 60 rpm, 70% maximal HR under normoxia) submaximal bicycle exercise. These authors reported that submaximal exercise under hypoxia (F_i_O_2_ = 14.5% and below) might be associated with an increase in blood lactate level, VE, HR, EF, and CO. These changes are an acute compensation response to reduced aerobic capacity owing to decreased oxygen delivery and utilization capacity under hypoxia. Regarding cardiac function, our study showed that HR was significantly higher in MH and LH groups than in the N group. This result is consistent with previous studies reporting that hypoxia reduces the ability to deliver and utilize oxygen to active muscles, but cardiac stress such as increase in HR raises the energy requirement during submaximal exercise with the same workload [7,10,26,28,29,30]. Unlike previous studies, the present study did not show changes in CO or EF during endurance submaximal exercise between environmental conditions. Considering that Moon et al. [26] observed higher CO and EF under severe hypoxia than under normoxia, no difference in CO and EF between LH, MH, and N conditions is considered a reasonable finding. However, considering that CO showed an increased tendency in the MH group compared to the N and LH groups in the present study, if the sample size was larger, it is considered that the statistical significance of CO would have been reached in response to reduced oxygen availability in active muscles following reduction in the oxygen-delivery capacity of the blood under hypoxia, as in most previous studies [7,10,26].

In addition, our study shows that blood lactate levels significantly increased in the MH group compared to the LH and N groups. Acute exposure to hypoxia results in sympathetic activation that raises blood epinephrine [31,32] and consequently lactate turnover in resting men and those engaged in submaximal exercises at given exercise power outputs [32,33]. Hypoxia also decreases metabolic and cardiac functions, which may increase the dependence on glycolysis—that is, anaerobic energy metabolism. This phenomenon induces an increase in VE, VCO_2_, RER, and blood lactate levels during exercise [26,34]. Endurance exercise under hypoxia decreases S_p_O_2_, resulting in an increase in the energy supply via the glycolytic system and consequently augmenting the blood lactate level in the active muscle [7,8,9]. In addition, the increase in VE during endurance exercise is indicative of an acid–base response by the bicarbonate buffer system that also occurs with an increase in blood lactate level [7,8,9]. Lühker et al. [35] investigated acid–base response differences during endurance exercise under normoxia and severe hypoxia (F_i_O_2_ = 12%, simulated 4500 m), and reported that severe hypoxia significantly decreased the pH and increased blood lactate levels during endurance submaximal exercise compared with normoxia. On the other hand, Sumi et al. [8] confirmed the effects of acid–base response differences on high-intensity interval exercise under moderate hypoxia (14.5% F_i_O_2_, simulated 3000 m) and normoxia in endurance athletes. These authors reported that high-intensity interval exercise under moderate hypoxia induced an increase in the blood pH, bicarbonate, and lactate levels compared with an equivalent level of exercise under normoxia. Although the acid–base response was not examined in our study, there was no significant difference in VE, VCO_2_, or RER between LH, MH, and N conditions. The discrepancy between previous studies is thought to be associated with the differences in hypoxic conditions, exercise type, and exercise intensity. Our results are considered reasonable because hypoxia the was not severe (more than simulated 4000 m) but light (simulated 2000 m) and moderate (simulated 3000 m), and the exercise type was not high-intensity interval exercise.

Hemorheological properties can help evaluate the oxygen delivery and utilization capacity in various subjects, for different exercise methods and exercise intensities, and between sexes [23,36,37,38]. In particular, hemorheological properties, including erythrocyte deformability and aggregation, are very important parameters because they are related to the surrounding microcirculation tissue and facilitate the exchange of oxygen and carbon dioxide [22]. In their study related to hypoxia and hemorheological properties, Grau et al. [39] investigated the impact of mild-to-severe hypoxia on human erythrocyte-nitric oxide synthase (NOS)-dependent nitric oxide production, protein S-nitrosylation, and deformability. These authors reported that the activation of erythrocyte-NOS decreased under mild hypoxia with an increase in hypoxia and erythrocyte deformability, which is influenced by erythrocyte-NOS activation, but surprisingly increased under severe hypoxia in vivo and in vitro. Moon et al. [21] reported that erythrocyte deformability was more reduced in healthy men under severe hypoxia (12.8% F_i_O_2_, simulated 4000 m and 11.2% F_i_O_2_, simulated 5000 m) than under normoxia, and was associated with a decrease in S_p_O_2_ concomitant with a compensatory increase in HR and a decrease in aerobic capacity, as reflected by an increase in blood lactate level. In addition, Lin et al. [40] confirmed that an acute bout of exercise under severe hypoxia (F_i_O_2_ = 12%, simulated 4500 m) increases erythrocyte aggregation and facilitates erythrocyte senescence in sedentary men. As such, most of the previous studies showed a decrease in erythrocyte deformability and an increase in erythrocyte aggregation during exercise under hypoxia compared to under normoxia, and these responses were confirmed to be prominent during exercise under severe hypoxia. Our study showed no significant differences between all trials in terms of erythrocyte deformability and aggregation. In the future, we believe that it is necessary to investigate erythrocyte deformability and aggregation under various conditions because the hemorheological properties are also greatly affected by hypoxic conditions, exercise type, exercise intensity, and exercise time.

## 5. Conclusions

The present study demonstrates that in comparison with endurance submaximal exercise under normoxia, endurance submaximal exercise under light (596 mmHg, simulated 2000 m) and moderate (526 mmHg, simulated 3000 m) hypobaric hypoxia induced greater metabolic (e.g., S_P_O_2_ and blood lactate level) and cardiac responses (e.g., HR). However, exercise under hypobaric hypoxia did not affect hemorheological properties, including erythrocyte deformability and aggregation, compared to exercise under normoxia.

## Data Availability

The data presented in this study are available on request from the corresponding author.
